# The vaginal microflora changes in various stages of the estrous cycle of healthy female dogs and the ones with genital tract infections

**DOI:** 10.1186/s12917-020-02710-y

**Published:** 2021-01-06

**Authors:** Edyta Golińska, Natalia Sowińska, Anna Tomusiak-Plebanek, Marlena Szydło, Natalia Witka, Joanna Lenarczyk, Magdalena Strus

**Affiliations:** 1 Jagiellonian University Medical College, Czysta 18, 31-121 Cracow, Poland; 2 University Center of Veterinary Medicine, University of Agriculture in Krakow, Cracow, Poland; 3 Animal Reproduction Unit, Department of Genetics and Animal Breeding, Faculty of Veterinary Medicine and Animal Science, Poznan University of Life Sciences PULS, Poznan, Poland; 4 Veterinary Clinic Biały Kieł, Białoruska 17a, 30-638 Cracow, Poland; 5 Veterinary Clinic Felis, Dworcowa 32, 32-620 Brzeszcze, Poland; 6 Veterinary Clinic Multivet, Feliksa Konecznego 6/12u, 31-216 Cracow, Poland

**Keywords:** Genital tract, Microflora, Inflammation, Probiotics, Bitch

## Abstract

**Background:**

Inflammatory diseases of reproductive tract in bitches are a common problem in veterinary practice. The inflammation can lead to serious health problems. Research to determine the correlation between the health status of females, phase of the cycle, age and bacterial flora of the genital tract has been ongoing for years, but the results obtained by individual authors are often contradictory.

**Results:**

A total of 39 dogs were included in this study. Ten were qualified to the 1st group with genital tract infections (8 in anestrus and 2 in proestrus) and 29 to the 2nd group without such infections (16 in anestrus, 9 in proestrus and 4 in diestrus). The most common bacterial isolates obtained from the vaginal tract of all dogs were *Escherichia coli, Staphylococcus pseudintermedius* and *Streptococcus canis.* The prevalence of Gram-negative rods (other than *E. coli*) was significantly higher in the group with genital tract infections versus healthy dogs. There was no presence of Chlamydiaceae, Chlamydia abortus and lactic acid-producing bacteria in tested swabs.

**Conclusions:**

Our study identified the most common bacteria in the genital tract of bitches. The total number of bacteria was almost the same in the healthy and infected dogs, as well as between the cycle stages. In our opinion, bacterial culturing of vaginal swab specimens from bitches without signs of genital disease is of little value. Furthermore, it should always be preceded by clinical examination and cytological examination of the vaginal epithelium.

## Background

Recurrent genito-urinary tract infections in bitches are a common problem in veterinary practice [1]. Vaginitis, both juvenile and adult-onset, infertility, abortion, foetus resorptions and mortality as well as cystic endometrial hyperplasia are among the most common disorders that might have a bacterial base [2–4]. As in the case of epithelium of other tissues which are in contact with external environment, like nasal cavity, oral cavity and alimentary tract, also vaginal epithelium and mucosa are colonized by physiological microflora [5]. Presence of bacteria, free or inside the epithelial cells, is frequently reported in vaginal smears of healthy dogs [6, 7]. Depending on the cycle stage from 50% do 100% of clinically healthy dogs are characterized by a vaginal bacterial population, usually mixed, consisting of both aerobic and anaerobic microorganisms [8–10], often opportunistic pathogens. It has been shown that normal vaginal microbiota appears to protect genito-urinary tract against potentially pathogenic bacteria by competing for nutrients or interfering with adhesion to epithelial cell receptors [1]. That’s why it is believed that genito-urinary disorders are often associated with changes in vaginal microflora [11, 12]. On the other hand it has been shown that bacterial species isolated from bitches with reproductive disorders do not differ significantly from those found in healthy bitches [10]. Kustritz suggested that reproductive tract infections are caused by overgrowth of normal local microbial flora, so it may be important for the outcomes of bacteriologic tests to be quantitative [9].

### The aim

Limited information is available about qualitative and quantitative composition of the natural vaginal microflora of healthy bitches and those with infections, and moreover, the results obtained by different authors are contradictory and therefore not conclusive. The aim of this study was to determine bacterial populations in various stages of the estrous cycle in the vagina of healthy dogs compared with the dogs with genital tract infections. This research will contribute to the understanding of possible oral supplementation of female dogs with non-pathogenic bacteria that colonize the normal vaginal flora of healthy female dogs.

## Results

A total of 39 dogs were included in this study. Ten were qualified to the 1st group with genital tract infections and 29 to the 2nd group without such infections (Table 1).

**Table 1:** Clinical groups of tested animals

Clinical groups	Healthy			With genital tract infections		
	*N* = 32			*N* = 10		
**Estrous phase**	Anestrus	Proestrus/Oestrus	Diestrus	Anestrus	Proestrus	Diestrus
	*N* = 16	*N* = 10	*N* = 5	*N* = 8	*N* = 2	*N* = 0

The lowest pH (5–5.5) values were observed in proestrus phase in individual studies in both groups. The average pH values were almost the same in the healthy and infected dogs, as well as between the cycle stages.

**Fig. 1:**
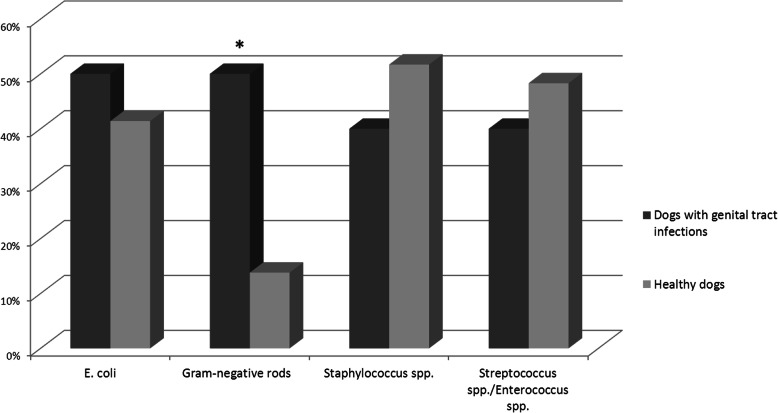
Vaginal bacterial populations of the dogs with genital tract infections compared to healthy dogs (*- *p* < 0.05)

The most common bacterial isolates obtained from the vaginal tract of all dogs were *Escherichia coli* (16/39), *Staphylococcus pseudintermedius* (15/39) and *Streptococcus canis* (13/39). The prevalence of Gram-negative rods (other than *E. coli*) was significantly higher (*p* < 0,05) generally in the group with genital tract infection (5/10) versus healthy dogs (4/29) (Fig. 1). *E. coli* was isolated from the vaginal tract of 50% dogs from the 1st group and of 41.4% dogs from the 2nd group. *Staphylococcus pseudintermedius* was isolated from 30% dogs in the 1st group and of 37.9% dogs from 2nd group and *Streptococcus canis* from 20 and 37.9%, respectively.

**Fig. 2:**
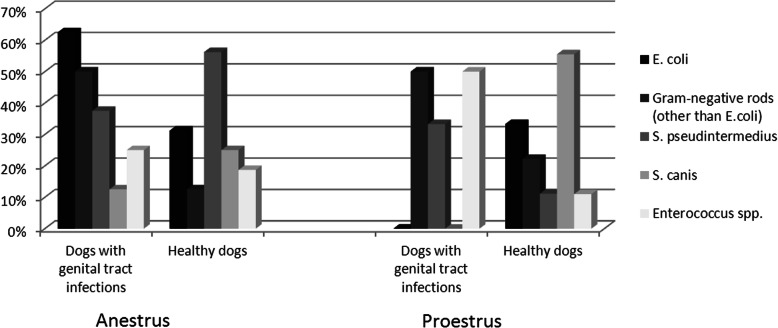
Vaginal bacterial populations in various stages of the estrous cycle

During the anestrus phase, *E. coli* was found in 5 out of 8 (62.5%) dogs with genital tract infections and in 5 of 16 (31.2%) of healthy dogs, and Gram-negative rods in 4 out of 8 (50%) and 2 out of 16 (12.5%), respectively. *S. intermedius* was isolated of 3 out of 8 (37.5%) dogs from the 1st group and from 9 of 16 (56.2%) in the 2nd group and *S. canis* in 1 out of 8 (12.5%) and 4 of 16 (25%), respectively. *Enterococcus spp*. in this phase was found in 2 out of 8 (25%) in the 1st group and 3 out of 16 (18.7%) in the 2nd group (Fig. 2).

During the proestrus phase, *E.coli* was not found in the 1st group and was isolated from 3 of 9 (33.3%) in the 2nd group and Gram-negative rods in 1 out of 2 (50%) and 2 out of 9 (22.2%), respectively (Fig. 2). *S. pseudintermedius* was found in 1 out of 3 (33%) dogs with genital tract infections and in 1 out of 9 (11.1%) healthy dogs. *S. canis* was not found in the vagina of dogs from the 1st group but it was isolated from 5 of 9 (55.5%) dogs in the 2nd group. *Enterococcus spp*. in this phase was found in 1 out of 2 (50%) in the 1st group and 1 out of 9 (11%) in the 2nd group.

The total number of bacteria was almost the same in the healthy and infected dogs, as well as between the cycle stages.

*Mycoplasma spp.* was found in all dogs from the 1st group. In the group of healthy dogs *Mycoplasma spp*. was found in 18 of 39 (62%) dogs (9/16 in anestrus, 7/9 in proestrus, 2/4 in diestrus) and *Mycoplasma canis* in 2 (22.2%) animals in proestrus and 1 (25%) in diestrus phase. There was no presence of Chlamydiaceae or *Chlamydia abortus* in tested swabs.

There was no presence of lactic acid-producing bacteria in the tested materials.

Based on statistical analysis, we revealed significant differences (*p* < 0.05) in the occurrence of *S. pseudintermedius* in the group of healthy dogs between the anestrus and proestrus phase.

There were no significant differences in the quantity of bacteria in any of the cycle stages and between healthy and infected groups.

## Discussion

This study was aimed to determine bacterial populations in various stages of the estrous cycle in the vagina of healthy dogs compared with the dogs with genital tract infections. The results suggest that there is a some variety in quantity and type of bacteria between different stages of the estrous cycle. The prevalence of common pathogens, such as *E. coli, S. pseudintermedius, S. canis* and *Enterococcus spp*., was similar between group of dogs with genital tract infections and the healthy ones. Almost the same results were obtained by Hutchins et al. [13]. Because bacteria are normal inhabitants of the lower reproductive tract, it is very difficult to determine if they are potentially pathologic or not, simply based on their presence. However, it is possible that a significant difference in the prevalence of specific pathogens such as *E. coli* would be identified in a larger study population. Only the presence of Gram-negative rods (other than *E.coli*) was significantly higher in the 1st group.

There was a large increase of *E.coli* and other Gram-negative rods population in anestrus phase in the vaginal swabs of dogs with infections. In contrast, *S. pseudintermedius* was most common in this phase in vaginal swabs of healthy dogs. Population of Gram-negative rods was almost the same in both estrus phases in the 1st group, whereas there was a large increase of *Enterococcus spp.* and large decrease of *E. coli* in proestrus phase.

*Streptococcus canis* is much more common in proestrus phase in healthy dogs, in contrast to *Enterococcus spp.* whose presence was more often noticed in dogs with genital tract infections. Therefore, it can be suspected that *Staphylococcus spp.* and *Streptococcus spp*. can act as protection against more dangerous pathogens, by competing for nutrients or interfering with adhesion to epithelial cell receptors. Or it could be due to the presence of blood in the vagina during proestrus.

We, like other research groups [14–16], observed that *Mycoplasma spp.* are common pathogens in the genital tract of tested groups. There was no relationship between the presence of *Mycoplasma* and the symptoms of infections.

This study shows a complete absence of *Lactobacillus* species in the genital tracts of tested dogs, unlike the vaginal microbiota of woman, where lactobacilli are predominant colonizers [17]. This result is in apparent disagreement with the studies of Delucchi [11] and Hurchins [12] groups. The cause for this is not clear. One of the possible explanations is the fact that in this study we used a sterile vaginal specula to collect swab samples, whereas in the previous studies an unguarded swab was used. This could cause contamination of the sample from the vestibule or vulva. However, Lyman et al. [16] using DNA sequencing observed that lactobacilli in the canine vagina represent only 0.03% of all bacterial populations. The same was observed in cow and ewe reproductive tracts [18].

In our study, the vaginal pH in dogs ranged from 5.0 to 8.1. This could be connected with the absence of lactic acid bacteria. In humans, where lactobacilli are predominant members of the microbiota, the normal vaginal pH is 4.5 or even lower.

Our own results, as well as results of other authors [7], pay particular attention to the important aspect of collecting knowledge in the field of vaginal microbiota as the starting point to find a way to effectively prevent urogenital infections.

## Conclusion

In conclusion, cultures of the vaginal canal will almost always result in growth of bacteria and interpretation of this growth is very difficult in terms of determining the need for treatment. In fact, bacterial culturing of vaginal swab specimens from bitches without signs of genital disease is of little value. It should always be preceded by clinical examination and cytological examination of the vaginal epithelium.

## Methods

### Samples collection

Client-owned dogs were carried to the Veterinary Clinic of the University Center of Veterinary Medicine JU-AU, University of Agriculture in Cracow, Poland in connection with reproductive problems, estrous monitoring, determination of mating date or routine gynaecological examination of breeding bitches. The owners were informed about the purpose of the study and gave their written consent for their dogs to participate in the study.

Samples for microbiology were collected from 39 bitches of different breeds, aged from 6 months to 10 years (mean 4.45 ± 2.4) (Table 1). The study included bitches in good shape only, showing no systemic or organ diseases (excluding the symptoms of vaginitis). Bitches undergoing any treatment were excluded. Animals were not given antibiotics for at least 2 weeks prior to collection.

Before samples’ collection, the veterinarian interviewed the animal owner and physical and gynaecological examinations of the bitch were performed.

Samples for microbiological examination were taken from the upper vault of the vagina, in the dorsal section, using a sterile swab and a sterile vaginal specula for bitches, model Hannover (Eickemeyer, Tuttlingen, Germany) of 150 mm in length and 5, 10 or 15 mm diameter (size adjusted to the bitch). The microbiological samples were delivered to the laboratory in the transport medium within 4 h from the collection.

In order to determine the phase of the cycle, cytological smears from the upper vault of the vagina of the bitches were taken with the use of a sterile swab moistened with 0.9% NaCl solution. After cytological preparation on a glass slide, the smear was fixed with Cytofix® (Samko, Klembów, Poland), subsequently smears were stained with a modified Wright Giemsa stain (Hemacolor® staining kit, Merck, Darmstadt, Germany). After drying, the smears were evaluated under the optical microscope Leica DM2500 (Leica, Wetzlar, Germany) at the magnification of 200× and 400×. Five types of epithelial cells were identified in the evaluated smear: basal cells, small intermediate cells, large intermediate cells, superficial cells and superficial cells without nuclei according to the classification proposed by Bowen [19]. In addition neutrophils, erythrocytes, bacteria, strands of mucus and cellular detritus were recognized in the smear.

### Qualification criteria for clinical groups

#### Vaginal pH measurement

The pH measurement was performed with the use of the MColorpHast™ indicator strips (Merck, Darmstadt, Germany) to which the tip of the vaginal speculum was applied immediately after removing it from the vagina. The pH was evaluated 2 s. after, according to the manufacturer’s recommendations.

#### Measurement of serum progesterone levels

5 ml of venous blood was drawn from each bitch. Progesterone serum level was tested by chemiluminescence using IMMULITE® 1000 apparatus (Healthineers Siemens, Erlangen, Germany).

#### Qualifications for clinical groups

Qualifications for clinical groups were made based on the medical history, clinical examination, cytological examination of the vaginal epithelium and the level of progesterone in the serum. First, the females were classified into groups with or without vaginitis based on the guidelines provided by Feldman and Nelson [1]. Subsequently the phase of the estrous cycle was determined.

Bitches with mucic, milky-white, yellowish or greenish vaginal discharge and the presence of numerous neutrophils in cytological smear were classified as animals with vaginitis (ill bitches, *n* = 10). Often in bitches with vaginitis, owners observed increased licking of the vulva by the animal and polydypsia/polyuria. Moreover, strands of mucus in cytological smears were visible. Bitches without vaginitis (healthy bitches, *n* = 29) had physiological vaginal discharge (from blood to straw coloured) or no discharge at all. The presence of few neutrophils in the cytological smear was acceptable only at the beginning of the proestrus phase, with simultaneous blood vaginal discharge and the diestrus phase, with no vaginal discharge.

In clinical terms, the whole cycle was divided into three periods: a period of heat or follicular phase, which consists of proestrus and estrus phases, the after-heat period, also called - the luteal phase, represented by the diestrus phase and the period of hormonal mute represented by the anestrus phase. The different phases of the cycle were determined on the basis of the guidelines described by Concannon [20].

Bitches in proestrus phase showed signs of increased estrogenization of the genital tract during the clinical examination, such as swelling and increased turgor of vulva (plump texture of the vulva), vaginal discharge (bloody or sero-bloody). Information from the medical interview with the owner described an increased interest of male dogs, with lacking or weak acceptance from the bitch. In the cytological picture, numerous epithelial cells were identified, from small intermediate cells through large intermediate cells, to superficial cells, mainly nucleated. In addition, numerous erythrocytes, and in the early proestrus single neutrophils (Table 2) were seen. Serum progesterone levels in the proestrus phase were below 1 ng / ml.

**Table 2:** Characteristics of the bitches used in the study

	Dog number	Age	pH	Level of progesteron (P4) in ng/ml	Total number of bacteria (c.f.u/ml)	Etiological factors (PCR)			
						Mycoplasma spp.	Mycoplasma canis	Chlamydiaceae	Chlamydia abortus
a) Dogs with genital tract infections									
**Anestrus**	**8**	**0.5**	6.3	<0,2	1,3x10^5	+	-	-	-
	**13**	**1**	6.0	<0,2	1x10^3	+	+	-	-
	**21**	**4**	6.6	<0,2	2,2x10^4	+	-	-	-
	**23**	**4**	6.6	0.78	2x10^2	+	-	-	-
	**30**	**4**	7.2	0.49	6x10^2	+	-	-	-
	**37**	**4**	6.9	<0,2	2x10^4	+	-	-	-
	**39**	**4**	6.6	0.91	4x10^3	+	-	-	-
	**40**	**4**	6.9	0.99	1x10^4	+	-	-	-
	**average**	**3.19**	**6.64**	**0.79**	**1,0x10^4**				
**Proestrus**	**16**	**1.5**	5.5	0.91	1x10^5	+	-	-	-
	**38**	**4**	8.1	0.69	2,8x10^3	+	-	-	-
	**average**	**2.75**	**6.8**	**0.8**	**3,4x10^4**				
b) Healthy dogs									
**Anestrus**	**1**	**4**	7.0	<0,2	1x10^3	+	-	-	-
	**2**	**3**	7.0	0.4	lack of growth	+	-	-	-
	**4**	**10**	7.0	0.27	2x10^4	+	-	-	-
	**9**	**3**	6.9	0.67	lack of growth	+	-	-	-
	**11**	**6**	6.9	0.42	2,2x10^4	-	-	-	-
	**12**	**2**	6.3	<0,2	1x10^4	-	-	-	-
	**14**	**6.5**	6.9	0.22	3x10^4	-	-	-	-
	**17**	**2**	6.9	<0,2	2,2x10^3	+	-	-	-
	**20**	**4**	6.6	0.42	1x10^3	+	-	-	-
	**24**	**3**	6.6	<0,2	1,1x103	+	-	-	-
	**27**	**4**	6.9	<0,2	1x10^5	+	-	-	-
	**28**	**4**	6.9	0.46	1x10^3	-	-	-	-
	**29**	**4**	6.9	0.95	2x10^2	-	-	-	-
	**32**	**4**	7.5	1.31	4,1x10^4	-	-	-	-
	**41**	**4**	6.6	<0,2	1,4x10^3	-	-	-	-
	**42**	**4**	7.2	<0,2	4,7x10^3	+	-	-	-
	**average**	**4.22**	**6.88**	**0.57**	**8,2x10^3**				
**Proestrus/Oestrus**	**6**	**4**	7.2	0.41	1x10^4	+	-	-	-
	**19**	**4**	6.3	24.9	1x10^5	+	+	-	-
	**22**	**4**	6.9	0.57	2,8x10^3	+	-	-	-
	**25**	**7**	5.0	0.2	lack of growth	+	-	-	-
	**26**	**3**	5.3	0.3	2x10^4	+	-	-	-
	**31**	**4**	7.2	0.24	6x10^2	-	-	-	-
	**33**	**4**	6.1	0.31	2x10^2	+	-	-	-
	**34**	**4**	5.5	25.4	5x10^3	+	+	-	-
	**35**	**4**	6.8	0.98	7x10^3	-	-	-	-
	**average**	**4.22**	**6.26**	**5.92**	1,1x10^4				
**Diestrus**	**3**	**10**	6.1	3.65	2x10^4	-	-	-	-
	**5**	**4.5**	6.0	1.95	1,1x10^4	+	-	-	-
	**10**	**4**	6.6	4.63	2,7x10^4	-	-	-	-
	**15**	**3**	7.2	38.6	1,5x10^4	+	+	-	-
	**average**	**5.38**	**6.48**	**12.21**	**6,1x10^4**				

Bitches in estrus phase performed sexual (acceptance) reflex, such as flagging of the tail (elevation of the tail away from the vulva and swaying of the hips from side to side) in response to touching the perineal region. In addition, in the clinical examination vulva was moderately swollen and had a pasty consistency (less turgid and more soft and flaccid than in proestrus). The vaginal discharge was still present, although it was lightly to straw coloured. In the cytological picture, only superficial cells, mainly without nuclei, and clear, “water-like” background was visible. The level of progesterone was above 1 ng / ml, although this was not a key eligibility criterion (Table 2).

Bitches in diestrus phase did not show the acceptance reflex, had no vulvar oedema or vaginal discharge. In cytological smear vaginal epithelial cells (of various types, mainly basal and intermediate cells) arranged in strands and isolated neutrophils were seen. The progesterone serum level was above 2 ng / ml (Table 2).

In anestrus phase, bitches, just like in diestrus phase, showed no acceptance reflex tolerance, in the clinical examination the vulva was not swollen and no discharge was recognized, but in cytological vaginal smears only a few epithelial cells, including mainly basal cells were seen. The level of progesterone was less than 1 ng / ml (Table 2).

#### Identification of vaginal bacterial populations

The vaginal swab was transferred from the transport medium (Amies, Deltalab, Barcelona, Spain) to 1 ml Schaedler’s broth (Becton, Dickinson and Company, Sparks, MD, USA) and agitated for 1 min. Serial decimal dilutions in the same broth were then made, and 100-μl aliquots were plated on standard media for cultivation: McConkey agar (Oxoid Ltd., Basingstoke, Hampshire, UK) for Enterobacteriaceae, Columbia blood agar (Oxoid) with 5% sheep blood for streptococci, BBL Enterococcosel agar (BD, Franklin Lakes, USA) for enterococci, Rogosa Agar (Merck, Darmstadt, Germany) for lactobacilli and Saboraud Agar (Merck) for Candida spp. The dilutions were then spread over plate surface by a glass rod and plates were incubated at 35 °C for a 24 h (for aerobic bacteria) or 48 h under microaerofilic condition (for lactobacilli). The morphology of the grown colonies were analysed under magnifying glass and several colony picks of each morphological type were subcultured on appropriate media and Gram-stained. After making subcultures, all colonies representing different morphotypes were counted on the plates showing appropriate colony density. Bacterial numbers were expressed as the log10 number of colony forming unit per 1 ml (c.f.u/ml). The subcultured colonies were further incubated and after checking for purity of the cultures, phenotypic identification was performed using commercial identification systems (API 20E, API50CH, APIStaph, APIStrep, API20NE (bioMerieux, l’Etoile, France).

#### Polymerase chain reaction-based gene detection of Chlamydiaceae and mycoplasma

The identification of the uncultivated microorganisms belonging to the Chlamydiaceae family, in particular the Chlamydophila abortus and Mycoplasma spp. including Mycoplasma canis was conducted using PCR. After plating the material taken from the upper vault of the vagina, the remaining material was used for DNA isolation using the commercial set Genomic Mini (A&A Biotechnology, Poland). PCR was performed based on previously described methods and primers [21–24]: CHYF (5′-GCC TAC CGG CTT ACC AAC-3′) and CHYR (5′- GGC GCA ATG ATT CTC GAT-3′) primers were used for *Chlamydiaceae* family identification, GPO-3 (5- GGG AGC AAA CAG GAT TAG ATA CCC T-3′) and MGSO (5′- TGC ACC ATC TGT CAC TCT GTT AAC CTC-3″) for *Mycoplasma* species, pmp-F (5′-CTC ACC ATT GTC TCA GGT GGA-3′) and pmp-R821 (5′- ACC GTA ATG GGT AGG AGG GGT-3′) for *Chlamydophila abortus* and MCF (5′- CAC CGC CCG TCA CAC CA-3′) and MCR (5′-CTG TCG GGG TTA TCT CGA C-3) for *Mycoplasma canis*. The PCR products were visualized on 1.5% agarose gel with ethidium bromide staining.

#### Statistical analysis

Statistical analyses were performed to demonstrate significant differences in the pH value as well as the presence of each microbial species within the tested group. pH values were compared using one-way ANOVA and frequency of microbial species was analysed using Fisher’s exact test. The significance level was set at *p* < 0.05.

## Data Availability

The data and materials are available from the corresponding author on reasonable request.

## Abbreviations

PCR: Polymerase Chain Reaction
